# Earthworms and Soil Pollutants

**DOI:** 10.3390/s111211157

**Published:** 2011-11-28

**Authors:** Takeshi Hirano, Kazuyoshi Tamae

**Affiliations:** 1 Department of Life and Environment Engineering, Faculty of Environmental Engineering, University of Kitakyushu, Kitakyushu, Fukuoka, 808-0135, Japan; 2 Division of Teacher Training, Faculty of Education and Culture, University of Miyazaki, Miyazaki, 889-2192, Japan; E-Mail: k-tamae@cc.miyazaki-u.ac.jp

**Keywords:** earthworm, metal, oxidative DNA damage

## Abstract

Although the toxicity of metal contaminated soils has been assessed with various bioassays, more information is needed about the biochemical responses, which may help to elucidate the mechanisms involved in metal toxicity. We previously reported that the earthworm, *Eisenia fetida*, accumulates cadmium in its seminal vesicles. The bio-accumulative ability of earthworms is well known, and thus the earthworm could be a useful living organism for the bio-monitoring of soil pollution. In this short review, we describe recent studies concerning the relationship between earthworms and soil pollutants, and discuss the possibility of using the earthworm as a bio-monitoring organism for soil pollution.

## 1. Introduction

It is well known that earthworms play an important role in the soil macrofauna biomass. They are extremely important in soil formation, principally by consuming organic matter, fragmenting it, and mixing it intimately with soil mineral particles to form water-stable aggregates [1]. In particular, the bioaccumulation ability of earthworms is essential for a bio-monitoring organism [2]. Therefore, the earthworm is likely to be an excellent organism for this purpose.

A bio-monitoring method would be appropriate to evaluate metal toxicity, because of its sensitivity and availability for unknown metabolites. Organisms such as fish, snails, and plants have been employed as bio-monitors [3–5]. Although this approach is useful and promising, it is also somewhat limited, because it could be available only for a specific combination of a living organism with certain substances. Hence, it is important to find appropriate living organisms as bio-monitors for each type of assessment.

Recent research has indicated that the earthworm is a candidate bio-monitor organism for soil pollutants, because it plays an important role in the soil macrofauna biomass. The species *Eisenia fetida* (Savigny, 1826, *E. fetida*) is most commonly used in ecotoxicology, and is recognized as a useful bio-monitor for testing the chemical toxicity of soil [6]. In particular, this species’ proximity to the soil contaminants is a merit for the analysis [7,8].

In this short review, we describe the possible use of the earthworm as a bio-monitoring organism for soil pollutants, by summarizing our previous work [9] and that of other researchers.

## 2. Effects of Metals on Earthworms

Earthworms have been indicated as a candidate bio-monitoring organism for soil pollutants. To establish a bio-monitoring system using earthworms, the effects of various chemical pollutants on earthworms have been studied. The accumulation of both natural and depleted uranium in earthworms was analyzed to evaluate the corresponding biological effects [10]. The study showed that no effects were observed in terms of mortality or weight reduction, but cytotoxic and genetic effects were identified at quite low natural uranium concentrations. Among metals, methyl mercury might be more easily absorbed by and accumulated in earthworms [2], suggesting that the earthworm is an ideal candidate for monitoring methyl mercury. Lee *et al*. also mentioned that metal bioaccumulation by earthworms could be used as an ecological indicator of metal availability [11]. On the other hand, metal pollution reportedly had no effect on earthworm communities [12].

Chemicals and ecological risk assessments should be based on the effects of the mixture, rather than those of a single compound. In this context, Natal-de-Luz *et al*. investigated the effects of sludge contaminated with chromium, copper, nickel, and zinc, and soils freshly spiked with the same mixture of metals, on *Eisenia andrei* [13]. They detected a decrease in the metal bioavailability for earthworms, promoted by the high organic matter content of the sludge. The binary mixture effects of cadmium and zinc on the mortality of *Aporrectodea caliginosa* were also investigated by Qiu *et al*. [14]. They reported that the effects of the cadmium and zinc mixtures on the mortality of *Aporrectodea caliginosa* were mainly antagonistic, and the magnitude of the antagonism was dependent upon both the relative concentrations of cadmium and zinc and their concentration magnitudes. The effects of combinations of metals and other chemical agents, such as pesticides, on earthworms were also investigated. Lister *et al.* studied the effects of a binary mixture of nickel and chlorpyrifos, an organophosphate insecticide, on Lumbricid earthworms, and found that both chemicals were rapidly accumulated to equilibrium [15]. Although the nickel uptake followed the same pattern as the single chemicals, the rates of chlorpyrifos uptake and elimination were faster, suggesting that a mixture of chemicals in soil might enhance the toxicity to organisms.

Taken together, an analysis using a single chemical compound does not necessarily reflect its toxicity for organisms in the environment. Therefore, when using earthworms as a bio-monitoring organism, careful attention should be paid to the target chemicals’ characteristics. A summary of the recently reported data about the effects of chemical agents in soil on earthworms is provided in Table 1.

**Figure 1.**
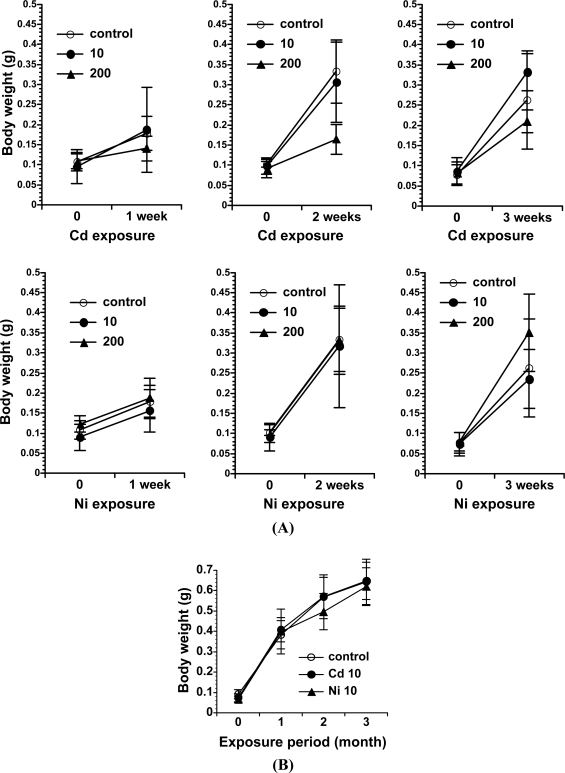
All *E. fetida* were weighed under wet conditions in the short-term **(A)** and the long-term **(B)** experiments. Each data point represents the mean of six *E. fetida*. The treatment of *E. fetida* with 200 μg Cd/g soil resulted in body weight loss, suggesting Cd-induced growth inhibition. In the long-term experiment, each data point represents the mean of 16 *E. fetida*, and no significant differences between any groups were observed. (Nakashima *et al*. [9], Copyright Elsevier.)

In terms of the relationship between metals and earthworms, cadmium has been extensively studied and is known to affect the earthworm’s physiological state, in terms of bioaccumulation [16], a slight decrease in weight [17], and oxidative DNA damage generation [9]. However, the earthworm is not useful for all chemical substances in soil. For example, nickel is not accumulated in the earthworm, and thus had no effects on growth, as shown in Figure 1 [9].

Among the factors that define the toxicological effects of metals on organisms are metal binding proteins, such as metallothioneins (MTs). MTs, cysteine-rich cationic proteins, play a critical role in preventing metal toxicity. Two MT isoforms (MT1 and MT2) were identified in the earthworm [24], and MT2 was increased in cadmium-exposed earthworms [25]. Stürzenbaum *et al*. demonstrated that the earthworm had the intrinsic capacity to efficiency sequester and compartmentalize cadmium via a metallothionein-mediated trafficking pathway, by using X-ray microanalysis, quantitative polymerase chain reaction, and immunohistochemical techniques [16]. Therefore, MT1/MT2 should be considered in the establishment of a bio-monitoring system using earthworms. These findings prompted us to assess the usefulness of earthworms as a bio-monitor. However, there are no established methods for using earthworms for this function.

## 3. Methods for the Detection of Metal Toxicity by Using Earthworms

In soil pollution, metal contamination is a critical event. To prevent metal toxicity, we must first understand the metal’s features and assess its toxicity. However, the methods used for the assessment of metal toxicity are often not sensitive enough, because metals can be toxic below the technical detection limits. To overcome this limitation, many research efforts have been made to develop detection techniques or assessment methods for metal contamination of soil. Furthermore, the toxic actions of metals sometimes depend on their metabolites generated in living organisms. Thus, adequate methods to assess metal toxicity are difficult to develop.

As we stated above, the earthworm is a candidate organism for the assessment of pollutants, but is not useful for all chemical substances in soil. Therefore, when using the earthworm as a bio-monitoring organism for soil pollutants, it is important to select the appropriate target substances. We have presented several methods using earthworms.

The analysis of earthworm reproduction is a useful method for the evaluation of chemical substances in the environment. The effects of cadmium and zinc on the reproduction of *Enchytraeus albidus* were investigated. Both metals reportedly affected the enchytraeids’ reproduction [26].

Nahmani *et al*. suggested that life cycle parameters, such as cocoon production and hatching rate, of *Eisenia fetida* were more sensitive to metal pollution than survival or weight change [27]. These studies revealed that the earthworm should be useful for the detection of the cytotoxic effects of certain types of chemical agents in soil, by selecting adequate parameters.

## 4. 8-Oxoguanine Generation in Metal-Treated Earthworm DNA

**Figure 2.**
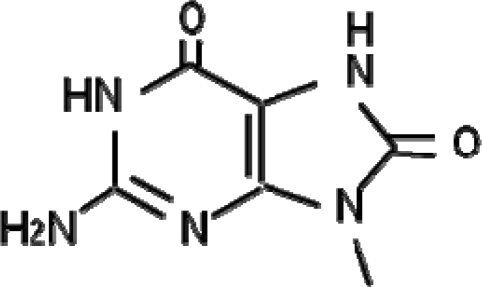
Structure of 7,8-dihydro-8-oxoguanine (8-oxo-Gua). 8-oxo-Gua is formed by hydroxylation of guanine at the C-8 position.

Quantitative and qualitative analyses of the oxidative DNA damage generated in living organisms are useful for the evaluation of the genotoxic effects of soil pollutants, because all aerobic organisms are constantly exposed to oxygen molecules. In particular, 7,8-dihydro-8-oxoguanine (8-oxoguanine, 8-oxo-Gua, Figure 2), a major form of oxidative DNA damage, may have an important role in carcinogenesis, because it causes the GC-to-TA transversion type point mutation [28–30]. 8-oxo-Gua is constantly generated in DNA and the nucleotide pool by reactive oxygen species (ROS), due to exposure to endogenous or exogenous factors.

**Figure 3.**
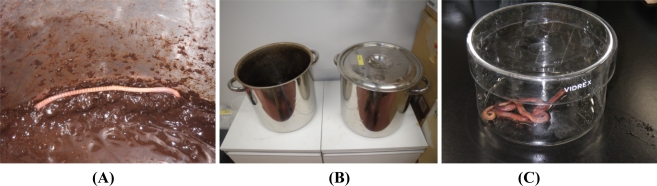
(**A**) *Eisenia fetida*; (**B**) *E. fetida* were kept in a 20 liter stainless steel tank; (**C**) *E. fetida* were maintained in a 600 mL glass container when they were exposed to heavy metals.

**Figure 4.**
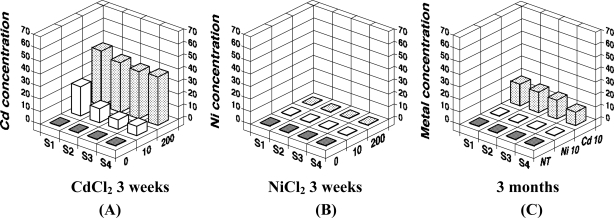
Heavy metal accumulation in *E. fetida*. Each data point represents the mean of three individuals. Heavy metal concentrations were measured by atomic absorption spectrometry, and are expressed as μg per body weight (data are modified from Nakashima *et al.* [9], Copyright Elsevier).

We recently analyzed the accumulation of 8-oxo-Gua in the DNA of *E. fetida* exposed to heavy metals, to determine if a method using earthworms as a bio-monitor would be useful for the assessment of soil mutagenicity [9]. We employed cadmium and nickel as test metals, because the carcinogenic potentials of cadmium and nickel have been established for humans and animals [31,32], and these metals are known to generate 8-oxo-Gua in DNA [33–36]. In the study, *E. fetida* were kept in a 20 liter stainless steel tank at an ambient temperature of 24 °C, using mold with skim milk as a food source until metal exposure [Figures 3(A,B)]. Three to six individuals were kept in a 600 mL glass container [Figure 3(C)] containing 50 g of soil with/without metals (CdCl_2_ or NiCl_2_). They were exposed to 10 or 200 μg metal/g soil for 1, 2, and 3 weeks or 10 μg metal/g soil for 3 months. As a result, we detected a high level of cadmium accumulation in *E. fetida* [Figures 4(A,C)]. On the other hand, no Ni accumulation was observed [Figures 4(B,C)].

**Figure 5.**
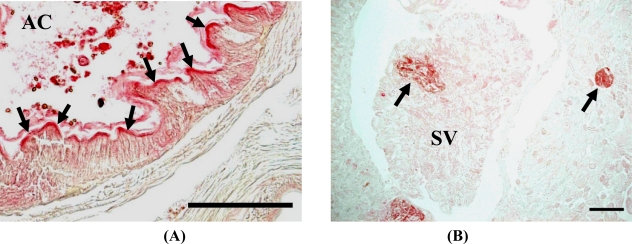
Immunohistochemical analyses of 8-oxo-dG accumulation in alimentary canal (AC) **(A)** and seminal vesicles (SV) **(B)** of *E. fetida* (S1) [data of (B) are modified from Nakashima *et al.* [9], Copyright Elsevier].

In addition, we observed positive 8-oxo-Gua staining in the gut epithelial layers in almost all samples [Figure 5(A)]. The metal absorption routes include the digestive system and the surface wall [37,38], but the main route is the digestive system. Since gut epithelial layers are frequently exposed to ROS, 8-oxo-Gua accumulation was constantly detected. Although the 200 μg cadmium-exposed *E. fetida* showed relatively stronger signals at 2 weeks in comparison to the others, almost all of the specimens showed positive signals in the gut epithelial layers, and the differences in the signal strength were too small to conclude that cadmium exposure increased 8-oxo-Gua accumulation in the organs. On the other hand, the positive signals in the seminal vesicles were clearly detected only in *E. fetida* treated with 10 μg of cadmium at 3 months [Figure 5(B)]. The seminal vesicles are considered as metallothionein (MT)-poor organs. Therefore, it seems reasonable to speculate that a lower level of MT expression is involved in the accumulation of cadmium-induced DNA damage.

Therefore, we would like to propose the merit of analyzing DNA damage generated in earthworms for the assessment of soil pollution. Besides analysis of 8-oxo-Gua generation, single-cell gel electrophoresis (comet) assay may be a useful tool for evaluation of genotoxic effects of pollutants on earthworms. Bonnard *et al*. indicated genotoxic effects of an industrially contaminated soil on *E. fetida* by using comet assay [39]. Although these studies suggested the utility of measuring DNA damage for evaluation, there are presently only a few reports about DNA damage in the earthworm [9,40].

## 5. Conclusions

Our previous data demonstrated the possible utility of using earthworms as a bio-monitoring organism, by measuring the oxidative DNA damage generated in the earthworms as a bio-monitoring method for assessing soil mutagenicity. However, many points remain unresolved. For example, this method would only be reliable for bio-accumulated metals, such as cadmium, but not for non-bio-accumulated metals, such as nickel, even if they generate 8-oxo-Gua. Furthermore, it is not clear how the pollution level of soil should be evaluated, based on the data from earthworms. Although several studies have been performed, no standardized method has been established, according to our knowledge. Further studies will be required to establish a broader bio-monitoring method using earthworms to assess soil pollution.

## Figures and Tables

**Table 1.** Recent reports on the effects of chemical agents in earthworms.

**Species**	**Chemical agents**	**Effects**	**References**
*Lumbricus rubellus*	Cd	Bioaccumulation	Stürzenbaum *et al*. (2004) [16]
*Lumbricus rubellus*	Cd and Cu	A slight decrease in weight.	Burgos *et al*. (2005) [17]
*Eisenia fetida*	Cd and Ni	Cd, but not Ni, accumulated and generated 8-oxoguanine in earthworm.	Nakashima *et al*. (2008) [9]
*Eisenia fetida*	Cu and ciprofloxacin (CIP)	Cu may be partly assimilated as Cu-CIP complexes I by earthworms, changing the bioavailability, subcellular distribution, and toxicity of Cu to earthworms.	Huang *et al*. (2009) [18]
*Lumbricus rubellus*	Pb and Zn	Accumulated in the posterior alimentary canal.	Andre *et al*. (2009) [19]
*Drawida* sp., *Allolobophora* sp., *Limnodrilus* sp.	Mercury	Mercury was mostly present in inorganic forms in earthworms. The bioaccumulation factors of methyl mercury from soil in earthworms were much higher than those of total mercury.	Zhang *et al*. (2009) [2]
*Lumbricus terrestris*	TiO_2_	An enhanced apoptotic frequency, which was higher in the cuticle, intestinal epithelium and chloragogenous tissue than in the longitudinal and circular musculature.	Lapied *et al*. (2011) [20]
*Eisenia fetida*	azodrin	Concentration-dependent changes in the morphology and the AChE activity.	Rao and Kavitha (2004) [21]
No description	polybrominated diphenyl ethers	Bioaccumulation	Sellström *et al*. (2005) [22]
*Eisenia fetida*	metalaxyl	Metalaxyl was rapidly assimilated by earthworms, and the bioaccumulation of metalaxyl was enantioselective, with preferential accumulation of the S-enantiomer.	Xu *et al*. (2011) [23]
